# Emotion-in-Motion, a Novel Approach for the Modification of Attentional Bias: An Experimental Proof-of-Concept Study

**DOI:** 10.2196/10993

**Published:** 2018-11-28

**Authors:** Lies Notebaert, Ben Grafton, Patrick JF Clarke, Daniel Rudaizky, Nigel TM Chen, Colin MacLeod

**Affiliations:** 1 Centre for the Advancement of Research on Emotion School of Psychological Science University of Western Australia Crawley Australia; 2 School of Psychology Curtin University Bently Australia

**Keywords:** attentional bias, anxiety disorders, experimental games

## Abstract

**Background:**

Individuals with heightened anxiety vulnerability tend to preferentially attend to emotionally negative information, with evidence suggesting that this attentional bias makes a causal contribution to anxiety vulnerability. Recent years have seen an increase in the use of attentional bias modification (ABM) procedures to modify patterns of attentional bias; however, often this change in bias is not successfully achieved.

**Objective:**

This study presents a novel ABM procedure, Emotion-in-Motion, requiring individuals to engage in patterns of attentional scanning and tracking within a gamified, complex, and dynamic environment. We aimed to examine the capacity of this novel procedure, as compared with the traditional probe-based ABM procedure, to produce a change in attentional bias and result in a change in anxiety vulnerability.

**Methods:**

We administered either an attend-positive or attend-negative version of our novel ABM task or the conventional probe-based ABM task to undergraduate students (N=110). Subsequently, participants underwent an anagram stressor task, with state anxiety assessed before and following this stressor.

**Results:**

Although the conventional ABM task failed to induce differential patterns of attentional bias or affect anxiety vulnerability, the Emotion-in-Motion training did induce a greater attentional bias to negative faces in the attend-negative training condition than in the attend-positive training condition (*P*=.003, Cohen *d*=0.87) and led to a greater increase in stressor-induced state anxiety faces in the attend-negative training condition than in the attend-positive training condition (*P*=.03, Cohen *d*=0.60).

**Conclusions:**

Our novel, gamified Emotion-in-Motion ABM task appears more effective in modifying patterns of attentional bias and anxiety vulnerability. Candidate mechanisms contributing to these findings are discussed, including the increased stimulus complexity, dynamic nature of the stimulus presentation, and enriched performance feedback.

## Introduction

### Attentional Bias Modification in Anxiety

We operate in a complex and dynamic world in which we are continuously confronted with an ever-changing stream of perceptual information. The limited capacity of our cognitive system means we can only attend to certain information, while other information is filtered out. Such filtering does not operate in the same manner across all individuals; however, it has become clear that there is a relationship between such attentional selectivity and individual differences in emotional vulnerability [1]. Specifically, research has shown that elevated anxiety vulnerability, whether indicated by elevated levels of trait anxiety or the presence of anxiety pathology, is associated with an attentional bias that favors the processing of negative information [2]. Moreover, studies that have manipulated patterns of attentional bias (using attentional bias modification [ABM] procedures) have shown that attentional bias causally contributes to anxiety vulnerability, as a change in attentional bias produces a consequent change in anxiety vulnerability [3,4-6].

The observation that ABM tasks delivered in the laboratory can exert a beneficial impact on anxiety responses to stressor has led a number of researchers to investigate whether extended exposure to such ABM training can reduce anxiety dysfunction in real-world settings. The most frequently used ABM procedure is based on the dot-probe task [7]. In this task, participants are briefly exposed to stimulus pairs, comprising 1 negative and 1 non-negative stimulus, before a small visual probe is presented, which participants are required to identify. A contingency between the position of the probes and the position of the negative stimuli is introduced, whereby probes are either always presented in the location where the non-negative stimulus was just displayed (encouraging the adoption of an attentional bias away from negative information) or else probes are always presented in the location where the negative stimulus was just displayed (encouraging the adoption of an attentional bias toward negative information). There is now a substantial body of evidence showing that such ABM tasks, configured to reduce attentional bias to negative information, can attenuate the symptoms of social anxiety disorder [8,9], generalized anxiety disorder [10,11], and subclinical obsessive-compulsive symptoms [12].

Although such encouraging findings highlight the potential clinical benefits of ABM procedures, it is important to recognize that in a number of ABM studies, the intended attentional training procedure has failed to affect emotional vulnerability [13-16]. Overall, meta-analyses show that the clinical effectiveness of the implementation of ABM procedures is small but nonetheless significant [17-21]. However, careful consideration of this literature suggests a clear pattern. In the studies where the intended ABM procedure successfully changed attentional bias, this produced a medium-sized and significant effect on emotional vulnerability. In contrast, in studies where the intended ABM procedure did not change attentional bias, no significant impact on emotional vulnerability was observed [22-26]. These results indicate that future research efforts should focus on developing more effective procedures than the dot-probe task to modify attentional bias [27-29]. Moreover, researchers have raised concerns about the suitability of the conventional probe-based training task for use with clinical cohorts because of its monotonous nature and low face validity [27].

### Gamification of Attentional Bias Modification Paradigms

In recent years, some investigators sought to adapt existing ABM procedures to make them more engaging by gamifying them. Gamification refers to the use of game design elements in nongame contexts [30]. Several levels can be discerned in the gamification of ABM tasks, from simply adding game elements to existing training tasks, to adding extrinsic rewards, adding intrinsic motivators, adding a game shell, to using an existing off-the-shelf game [31]. It is thought that maintaining a close connection with validated ABM tasks and adding intrinsic motivation and a game shell may be optimal for ABM gamification [31].

Existing gamified training protocols have been variants of the original probe approach; however, all these protocols share in common the limitation that they seek to train attentional selectivity using very simple static displays that typically present only 1 or 2 stationary emotional stimuli [28,32-36]. This contrasts markedly with real-world settings, which generally require individuals to engage in patterns of attentional scanning and tracking within a complex and dynamic environment [37]. In addition, these studies have been hampered by design limitations. In some cases, these studies have lacked a control condition [33,34]. Moreover, most of these novel procedures have been delivered in studies that afford no opportunity to compare their efficacy with that of the conventional probe-based ABM approach. Without such a comparison, it remains unknown whether a novel ABM task can achieve attentional bias change under conditions where the conventional probe-based ABM task fails to do so or whether a novel task can produce change in anxiety vulnerability when such change is not elicited by a conventional probe-based ABM approach.

These studies did, however, incorporate different gamification and other (nongame) elements to enhance engagement with the tasks to improve their effectiveness in modifying attentional bias [36,38]. Some studies have included *motivating feedback* or *goal metrics*, in the form of real-time visual performance feedback or points [32,35] or block-by-block feedback on performance [28]. Others have implemented more *elaborate displays* or a *game-shell* to increase intrinsic motivation [33,35,39-43]. Another element thought to increase intrinsic motivation is *goals-directed learning*, which directs players to particular goals to increase targeted skills (eg, through instructing participants to attend to positive information) [44]. However, despite inclusion of these gamification elements, the majority of these studies continue to rely on either relatively sparse or mostly static stimulus displays.

### This Study

The objective of this study was to develop and evaluate a novel candidate ABM procedure designed to modify attentional selectivity within a task setting that, like most real-world contexts, requires participants to selectively distribute attention while processing a complex and dynamic emotional environment. The task required participants to search for and track 1 particular target stimulus presented on screen among multiple moving distractors, based on its emotional valence. This screen display thus realizes the *elaborate display* gamification element, as it presents a large number of stimuli that dynamically move across the whole screen. The task also incorporated the gamification elements of motivating feedback through a game-by-game high score that participants were encouraged to try to beat as well as the element of goals-directed learning, as participants were explicitly instructed to track 1 particular emotional expression. The task was built upon the same principles as the original probe-based ABM task, in which the training condition was designed to increase attentional bias toward positive information, and the task performance would be improved to the extent that participants adopted a more positive attentional bias [6]. Similarly, in the corresponding training condition of the gamified task, performance will improve to the extent participants allocate attention to positive stimuli.

Our primary aim was to evaluate the capacity of this new candidate ABM procedure, which we have labeled the Emotion-in-Motion task, to induce a group difference in selective attentional responding to negatively and positively valenced information and to causally impact anxiety vulnerability, as evidenced by the strength of state anxiety responses to a controlled laboratory stressor. We also delivered the conventional probe-based ABM procedure to a separate cohort of similar participants under equivalent laboratory conditions. This conventional probe-based ABM task does not include any of the gamification elements introduced in the Emotion-in-Motion task. Specifically, there is no elaborate display (only 2 static images are presented), no motivating feedback after each block (only trial-by-trial feedback), and no goals-directed learning (participants are simply instructed to discriminate the identity of a probe). We chose to compare our novel Emotion-in-Motion task with the probe-based ABM task, as this is the procedure most commonly used in studies aiming to modify patterns of attentional bias [17,18,21].

This study design will enable us to determine (1) whether both the conventional probe-based ABM task and this new, complex, dynamic Emotion-in-Motion ABM task produce a group difference in attentional bias in line with the allocated attentional training condition, (2) if both tasks prove capable of so modifying attentional bias, whether the Emotion-in-Motion ABM task impacts attentional bias to an equal or greater degree than does the conventional probe-based ABM task, (3) whether both the conventional probe-based ABM task and this new, complex, dynamic Emotion-in-Motion ABM task serve to induce a group difference in anxiety vulnerability as a function of allocated training condition, and (4) if both tasks prove capable of so influencing anxiety vulnerability, whether the Emotion-in-Motion ABM task impacts anxiety vulnerability to an equal or greater degree than does the conventional probe-based ABM task.

## Methods

### Participants

A total of 129 undergraduate students at the University of Western Australia completed the study. In line with previous research, participants who did not show an elevation in state anxiety in response to the intended stressor were excluded before analyses [28]. This led to the exclusion of 19 participants, with 110 participants remaining. Participant characteristics are shown in Table 1.

### Conventional Probe-Based Attentional Tasks

Overall, 55 participants completed the conventional probe-based bias training and assessment tasks. These participants were randomly assigned to either an attend-positive or attend-negative training condition. Participants assigned to these 2 conditions of the probe-based tasks did not differ significantly in age, trait anxiety scores, or gender (all *P*>.05).

### Emotion-in-Motion Attentional Tasks

Overall, 55 participants completed our novel Emotion-in-Motion bias training and assessment tasks. Participants were randomly assigned to either an attend-positive or attend-negative training condition. Participants in these 2 conditions of the Emotion-in-Motion tasks did not differ significantly in age or trait anxiety scores (both *P*>.05). These 2 groups did differ significantly in gender ratio, *P*=.03, with a higher proportion of males in the attend-negative condition than in the attend-positive condition. Consequently, we considered gender ratio as a covariate in our analyses of the data, which provided reassurance that observed effects of this experimental manipulation remained evident when this group difference in gender ratio was accounted for.

**Table 1:** Age, gender, and trait anxiety scores (using the Spielberger Trait Anxiety Inventory) for participants completing the conventional probe-based and the Emotion-in-Motion attentional tasks in each of the 2 training conditions.

Condition		Age, mean (SD)	Gender, female/male	Trait anxiety, mean (SD)
**Conventional probe tasks**				
	Attend-negative condition (N=27)	19.33 (2.86)	14/13	38.44 (8.14)
	Attend-positive condition (N=28)	19.50 (2.60)	19/9	41.18 (11.08)
**Emotion-in-Motion tasks**				
	Attend-negative condition (N=28)	19.78 (3.62)	17/10	47.18 (8.18)
	Attend-positive condition (N=27)	18.50 (0.95)	23/3	43.22 (9.24)

### Materials

#### Attentional Tasks Stimuli

The face stimuli for the attentional tasks were selected from the Karolinska Directed Emotional Faces stimulus set [45]. These images were cropped to show only the face and the neck. The face stimuli for the training tasks were photos of 32 individuals, half of them were female and half were male. For the assessment tasks, photos of 8 different individuals were selected, half of them were male and half were female. There were 2 photographs of each individual, 1 in which they depicted a happy expression and 1 in which they depicted an angry expression. Each photograph was 258 pixels (width) by 323 pixels (height). The stimuli were the same size in the Emotion-in-Motion and probe-based tasks. For the Emotion-in-Motion training task, the 32 identities were grouped into 8 stimulus subsets, each containing the photos of 8 identities, 4 female and 4 male. Each stimulus subset was used in 1 of the 8 blocks delivered in this Emotion-in-Motion task.

#### Emotion-in-Motion Attentional Bias Modification Task

The aim of this task was to induce, in a complex and dynamic task environment, selective attending to angry or happy faces, depending on the assigned training condition. To provide readers with a first-hand impression of this Emotion-in-Motion task, the task can be viewed on the Web [46].

The Emotion-in-Motion ABM task consisted of 8 3.5 min blocks or *games*. During each block, 8 placeholder rectangles moved dynamically around the screen over a black background. Each rectangle contained an image of a face, each with a different identity. At all times, the target rectangle displayed a face with an emotional expression that differed from the emotional expressions displayed by the faces in all 7 other rectangles on screen, and participants were required to attend to and track this rectangle. In the *attend-negative* condition, the target rectangle displayed a face with an angry expression, whereas the other rectangles displayed faces with happy expressions. In the *attend-positive* condition, the target rectangle displayed a face with a happy expression, whereas the other 7 rectangles displayed faces with angry expressions. Participants were instructed to find the target rectangle and track it using the mouse cursor. All the rectangles, including the target, constantly switched faces. Participants were instructed to keep tracking the target rectangle (ie, depicting the single face with the expression differing from that of the other 7 faces) even when the face presented within changed, as long as the emotional expression of the face presented remained the same (ie, when the face in the target rectangle switched to a different identity, participants were required to keep tracking the rectangle as long as the emotional expression of the new face was the same as the emotional expression of the previous face). At random intervals, the emotional expression of a target face would change in addition to its identity, at which point this ceased to be the target rectangle. At that same moment, 1 of the other rectangles would assume a face depicting this emotion, and thus identifying it as the (new) target rectangle. At these points, participants had to quickly find the new target rectangle and start tracking it.

At the start of a block, each face remained constant for the first 2000 milliseconds. Thereafter, individual faces within a rectangle switched to a different identity (but same expression) randomly at any point between 1 and 2000 milliseconds throughout the block. Within each block, the target rectangle switched (thus, an expression switch occurred) 60 times, at random intervals of 5 to 10 seconds. All 8 rectangles moved with different randomly determined trajectories, at a randomly determined speed of between 30 and 50 pixels per 100 milliseconds. Thus, although the rectangles moved at different speeds, each rectangle’s speed was constant within a game. The rectangles bounced off the screen edges and other stimuli they contacted at an angle of reflection that matched their angle of incidence. The target rectangle was never indicated; however, when the mouse cursor was correctly located in the position of the current target rectangle, this cursor disappeared *behind* the rectangle (to not obscure the face presented within) and remained hidden as long as the participant kept it on target.

The onset of each block was preceded by a 3-second countdown presented in the center of the screen. At the end of each block, participants were presented with a tracking score (ie, the percentage of time during that game they were tracking the target rectangle), a switching score (ie, the average speed with which the participant was able to shift their cursor to the next target rectangle), and a total score for that block (generated by combining the tracking score and the switching score). The screen also displayed the participant’s highest prior (total) score. Participants were instructed that they would play several games of this task and were encouraged to beat their current high score in each successive game.

### Emotion-in-Motion Attentional Bias Assessment Task

The training contingency was removed from the Emotion-in-Motion training task to create the assessment task used to reveal the impact of this training on attentional selectivity. Thus, participants were required to track a rectangle displaying a face with a happy expression (among 7 rectangles displaying faces with angry expressions) on half of the blocks and to track a rectangle displaying a face with an angry expression (among 7 rectangles displaying faces with happy expressions) on the other half of the blocks. This assessment task delivered 12 short blocks, each of which contained 5 target switches, resulting in a total of 60 target switches across the assessment task. In 6 of these blocks, the target rectangle displayed a face with an angry emotional expression, and in 6 blocks, the target rectangle displayed a face with a happy emotional expression. The order of these block conditions was randomly determined, with the constraint that a maximum of 2 consecutive blocks could have a target with the same valence. Each block started with a 5-second countdown.

To obtain a measure of attentional bias to negative information, an attentional bias index (ABI) was computed by subtracting the average tracking score a participant obtained in blocks where targets were happy faces from the average tracking score the participant obtained in blocks where targets were angry faces. Therefore, a higher positive score on this index reflects greater attention to negative information, as it represents more successful tracking of angry than of happy faces.

### Other Experimental Tasks

The Trait Anxiety Assessment, conventional probe-based ABM, and assessment tasks as well as the anxiety reactivity assessment task are described in Multimedia Appendix 1.

### Procedure

Participants were tested individually in a sound-attenuated room. Once informed consent from participants had been obtained, participants were instructed to sit at a comfortable viewing distance from the computer screen (approximately 60 cm), were given instructions, and completed the first assessment task. After completion of the training task, they completed the original assessment task again. Next, participants completed the anxiety reactivity assessment task containing an anagram stressor task preceded and followed by a measure of state anxiety. At the end of the session, participants were debriefed about the purpose of the study. The entire experimental session lasted about 1 hour. This study was approved by the University of Western Australia’s Human Research Ethics Committee, protocol RA415243.

## Results

### Impact of Attentional Training Procedure on Attentional Bias

The criteria to identify outliers are described in Multimedia Appendix 1. The ABI scores obtained before and after the training task are shown in Table 2.

### Attentional Impact of Conventional Probe-Based Training

Application of the outlier criteria led to the exclusion of 4 participants (2 in the attend-positive training condition). To examine whether the conventional probe-based training task was capable of modifying attentional bias, a mixed-methods analysis of variance (ANOVA) was performed with the within-subjects factor attentional assessment point (pretraining assessment, posttraining assessment) and the between-subjects factor training condition (attend-positive training, attend-negative training). The ABI scores obtained by participants who completed this conventional probe-based training task served as the dependent variable.

Results showed neither a significant main effect of attentional assessment point, *F*_1,48_<1, nor of training condition, *F*_1,48_=2.246, *P*=.14. Most importantly, the critical interaction between attentional assessment point and training condition fell short of significance, *F*_1,48_=3.018, *P*=.09, η^2^_p_=.059.

### Attentional Impact of Emotion-in-Motion Training

Application of the outlier criteria led to the exclusion of 3 participants (1 in the attend-positive training condition). To determine whether our novel Emotion-in-Motion attentional training procedure was effective in modifying attentional responding to negative information, the ABI scores obtained by participants who completed this task were subjected to a mixed-design 2x2 ANOVA that again considered the within-group factor attentional assessment point (pretraining assessment vs posttraining assessment) and the between-group factor training condition (attend-positive training vs attend-negative training). This analysis revealed a significant main effect of training condition, *F*_1,50_=4.602, *P*=.04, η^2^_p_=.084, subsumed within a higher-order interaction of attentional assessment point x training condition, *F*_1,50_=5.629, *P*=.02, η^2^_p_=.101. At pretraining, there was no significant difference between the ABI scores obtained by participants in the attend-positive training condition and participants in the attend-negative training condition, *F*_1,50_<1. In contrast, at posttraining, participants in the attend-negative training condition showed significantly higher ABI scores as compared with participants in the attend-positive condition, *F*_1,50_=9.903, *P*=.003, Cohen *d*=0.87. Although the change in attentional bias from pre- to posttraining fell short of significant for participants in the attend-negative training condition, *t*_25_=−1.162, *P*=.26, Cohen *d*=0.229, there was a significant change from pre- to posttraining for participants in the attend-positive training condition, *t*_25_=2.114, *P*=.045, Cohen *d*=0.415. Overall, this pattern of results confirms that the 2 training conditions exerted a differential impact on attentional bias to negative information, and the direction of the observed attentional training effects was as expected. When controlling for the gender, by adding this as a covariate, this interaction between attentional assessment point and training condition remained significant, *F*_1,43_=4.393, *P*=.04, η^2^_p_=.087.

**Table 2:** Attentional bias index scores pre- and posttraining for participants who completed the conventional probe-based attentional bias training and assessment tasks or the Emotion-in-Motion attentional bias training and assessment tasks in either the attend-positive training condition or the attend-negative training condition.

Assessment point		Attend-positive condition, mean (SD)	Attend-negative condition, mean (SD)
**Conventional probe training**			
	ABI^a^ pretraining	−5.669 (50.752)	−3.116 (29.591)
	ABI posttraining	−15.101 (45.33)	11.002 (36.9)
**Emotion-in-Motion training**			
	ABI pretraining	0.449 (6.041)	1.011 (5.968)
	ABI posttraining	−2.739 (6.545)	2.445 (5.261)

^a^ABI: attentional bias index.

**Table 3:** State anxiety scores pre- and postanagram stressor for participants who previously completed the conventional probe-based attentional bias training task or the Emotion-in-Motion attentional bias training task in either the attend-positive training condition or the attend-negative training condition.

Assessment point		Attend-positive condition, mean (SD)	Attend-negative condition, mean (SD)
**Conventional probe training**			
	State anxiety pretraining	30.680 (10.858)	26.200 (12.176)
	State anxiety posttraining	43.600 (9.734)	40.440 (11.623)
**Emotion-in-Motion training**			
	State anxiety pretraining	31.292 (10.149)	29.541 (11.699)
	State anxiety posttraining	39.458 (11.026)	41.458 (10.879)

### Impact of Attentional Training Procedure on Anxiety Vulnerability

The state anxiety scores obtained using the analog mood scale given before and after the final anagram stressor are shown in Table 3.

### Emotional Impact of Conventional Probe-Based Training

Application of the outlier criteria on participants included in the attentional bias assessment analyses led to the additional exclusion of 1 participant (in the attend-positive condition). To examine whether the 2 training conditions had a differential impact on anxiety reactivity, state anxiety scores were subjected to a mixed-methods ANOVA with the within-subjects factor state anxiety assessment point (prestressor assessment vs poststressor assessment) and the between-subjects factor training condition (attend-positive training vs attend-negative training). Results showed a significant main effect of state anxiety assessment point, *F*_1,48_=159.991, *P*<.001, indicating that state anxiety increased from before the anagram stressor (mean 28.440, SD 11.639) to after the anagram stressor (mean 42.020, SD 10.729). However, neither the main effect of training condition, *F*_1,48_=1.664, *P*=.20, nor the critical interaction between state anxiety assessment point and training condition, *F*_1,48_=.378, *P*=.54, η^2^_p_=.008, were significant.

### Emotional Impact of Emotion-in-Motion Training

Application of the outlier criteria on participants included in the attentional bias assessment analyses led to the additional exclusion of 4 participants (2 in the attend-positive training condition). The same 2x2 ANOVA as reported above was conducted on state anxiety scores to examine whether in participants who completed the Emotion-in-Motion training procedure, the 2 training conditions had a differential impact on anxiety reactivity. This analysis revealed a significant main effect of state anxiety assessment point, *F*_1,46_=125.99, *P*<.001, η^2^_p_=.73, again reflecting the fact that state anxiety increased from before the stressor (mean 30.58, SD 10.87) to after the stressor (mean 40.54, SD 10.88). This main effect was now subsumed within a significant two-way interaction of state anxiety assessment point and training condition, *F*_1,46_=4.39, *P*=.04, η^2^_p_=.09. When controlling gender, by adding gender as a covariate, this interaction between state anxiety assessment point and training condition remained significant, *F*_1,43_=4.638, *P*=.04, η^2^_p_=.097.

Follow-up *t* tests revealed that immediately following the attentional training procedure but before the anagram stressor experience, participants who had received the 2 training conditions did not differ in their levels of state anxiety, *F*_1,46_=.31, *P*=.58, η^2^_p_=.01. Participants in each Emotion-in-Motion attentional training condition responded to this stress manipulation by displaying an elevation in anxious mood state (attend-positive training: *F*_1,23_=55.84, *P*<.001, η^2^_p_=.71 vs attend-negative training: *F*_1,23_=70.56, *P*<.001, η^2^_p_=.76). However, the magnitude of the elevation in state anxiety evoked by this stressor was significantly attenuated in those participants who had received the Emotion-in-Motion attend-positive attentional training compared with those participants who had received the Emotion-in Motion attend-negative attentional training condition (mean 8.17, SD 5.35 vs mean 11.92, SD 6.94; Cohen *d*=0.60). Thus, those participants who had been exposed to the Emotion-in-Motion task training contingency designed to reduce attentional bias to negative information subsequently came to display relatively attenuated elevations of anxious mood state in response to the anagram stressor experience compared with participants who had been exposed to the training condition designed to increase attentional bias to negative information. In addition, the elevation in anxiety in the positive training condition of the Emotion-in-Motion task (mean 8.17, SD 5.35) was significantly smaller than the elevation in state anxiety in the positive training condition of the conventional probe-based training task (mean 12.92, SD 8.83), *t*_47_=2.35, *P*=.02, Cohen *d*=0.67.

## Discussion

### Principal Findings

The objective of this study was to develop and evaluate a novel ABM procedure intended to systematically alter selective attentional responding to emotional information in a complex and dynamic task environment. Our results showed that our novel Emotion-in-Motion training procedure succeeded in modifying patterns of attentional bias, as intended. Moreover, the participants who were allocated to the attend-positive condition of the Emotion-in-Motion attentional training task showed reduced anxiety reactivity to the subsequent lab-based stressor as compared with participants who were allocated to the attend-negative condition of this task. These results suggest that our novel attentional training task appeared capable of modifying both patterns of attentional bias and causally influencing anxiety vulnerability.

A subsidiary aim was to permit comparison with the conventional probe-based attentional bias training procedure. Under equivalent laboratory conditions, the conventional probe-based attentional training approach failed to induce differential patterns of attentional bias, and the 2 probe-based training conditions did not lead to participant differences in anxiety reactivity to the subsequent stressor. In recent years, several studies (including 3 out of our lab) have reported similar failures of the conventional probe-based attentional training task to successfully modify patterns of attentional bias [28,47-51]; therefore, it is reasonable to conclude that the probe-based ABM procedure may be a nonoptimal way of achieving bias change.

### Candidate Explanations for the Effectiveness of the Emotion-in-Motion Task

In reflecting on the reasons for the capacity of our novel Emotion-in-Motion paradigm to induce differential patterns of attentional bias, under conditions where the conventional probe-based training did not, several candidate factors can be considered. First, the Emotion-in-Motion task presents 8 stimuli simultaneously, whereas the conventional probe task displays only 2 stimuli. There is some evidence that attentional bias is more pronounced when assessed using visual displays that contain more stimuli [52,53], but as yet, it is unknown whether more robust ABM effects can be obtained using paradigms that present more stimuli. Although some training procedures that involve more complex stimulus displays already exist [41,54], so far no direct comparison between the effectiveness of training tasks using simple versus complex stimulus displays has been made. In future research, the Emotion-in-Motion paradigm can be easily be adapted to present simple displays (eg, 2 rectangles) versus complex displays (eg, 8 rectangles), to enable such comparison.

A second candidate factor that could have contributed to the findings observed with the Emotion-in-Motion approach is the dynamic nature of the stimulus presentation. In the Emotion-in-Motion task, all stimuli move dynamically around the display, whereas in other attentional training paradigms, stimuli are presented in a static manner. It is possible that the dynamic nature of Emotion-in-Motion enhanced concentration and engagement with the task, thereby increasing its capacity to deliver the intended attentional bias change. In future research, the potential contribution of this dynamic component could be examined by contrasting task variants that employ the present dynamic approach with variants that instead present the same number of stimuli in static grid.

A third candidate reason for its efficacy may be the provision of enriched performance feedback in the Emotion-in-Motion task compared with the rudimentary trial-by-trial error feedback given in the conventional probe-based attentional training task. Moreover, block feedback in the Emotion-in-Motion task was encouraging, whereas trial-by-trial feedback in the probe-based task penalized participants for making errors by delaying the next trial for 3 seconds, which may have elicited increased negative mood. Block feedback of the type delivered in the Emotion-in-Motion task has been shown to enhance learning in simple repetitive tasks [55], whereas negative mood has been shown to impair learning [56]. As such, this difference in feedback may have also contributed to enhanced performance in the Emotion-in-Motion task. Future research could further examine the contribution of enriched performance feedback to the efficacy of ABM procedures by comparing conventional probe-based training with and without such block feedback or by manipulating whether or not the presently provided block feedback is delivered within the Emotion-in-Motion task.

Moreover, in the conventional probe-based training task, images depicting different emotional expression of the same identity were paired, whereas in the Emotion-in-Motion task, each image depicted a different identity. As such, participants performing the probe-based training only needed to discriminate emotional expression on the same person, whereas participants performing the Emotion-in-Motion tasks needed to discriminate emotional expressions between different identities. There is some evidence to suggest that emotion classifications are affected by variations in identity [57]. It is, therefore, possible that this increased demand on emotion classification contributed to the Emotion-in-Motion task being more challenging. The more challenging emotion classification, enhanced performance feedback, as well as the complex and dynamic nature of the task could have resulted in greater engagement with the Emotion-in-Motion task, relative to the conventional probe task. Task engagement can be conceptualized as a combination of energy, motivation, and concentration and can be measured using self-report as well as through task performance indicators [58]. In the Emotion-in-Motion task, we did not obtain self-report measures of task engagement, and the difference in the nature of the tasks leaves us unable to compare performance indicators of engagement. However, future research may usefully examine whether individuals show a difference in engagement with the Emotion-in-Motion task relative to the probe task and whether task engagement moderates the procedures’ impact on attentional bias and anxiety vulnerability [36].

An additional difference between the 2 training procedures concerns participants’ responses. The tracking response required in the Emotion-in-Motion task is continuous, whereas the probe task only requires a response every couple of seconds. It is likely that as a result, participants in the Emotion-in-Motion task spend more time attentionally engaged with the target valence (positive or negative, depending on training condition) as compared with participants in the conventional probe task. However, it is also possible that this continuous response would be harder to sustain over time as it is more motorically demanding. As such, future research may usefully examine the acceptability of this response format in multi-session training designs. It is also relevant to note that in the Emotion-in-Motion task, the mouse cursor only disappears behind the target rectangle. As such, it is possible that participants could ignore the content of the rectangle and simply see on which rectangle the mouse cursor would disappear. Although given the speed and complexity of the task this strategy is unlikely to have occurred, importantly, this strategy would have reduced the efficacy of the attentional training. Future research could, therefore, evaluate whether modification to the task (eg, making the cursor disappear behind every image) would further increase the effectiveness of the task. It will be important to establish, however, whether such modifications that render participants’ awareness of the position of the cursor more uncertain cause unwanted frustration or disorientation in participants.

### Strengths and Limitations

It is important to consider the potential limitations of this study. One such limitation is that the capacity of the Emotion-in-Motion training task and the capacity of the conventional probe-based training task to modify attentional bias were each established using a different method of assessing attentional bias. For both training tasks, the assessment approach involved delivering the same task but with the training contingency removed. This design critically allows for comparable demonstration of near transfer across the 2 training tasks. However, it does preclude direct comparison of attentional bias change observed in response to each of these 2 candidate attentional training approaches. It is possible, for example, that the assessment version of the Emotion-in-Motion task is more sensitive to individual differences in attentional bias than the probe-based attentional bias assessment task (of potential relevance to this, note that the SDs for Emotion-in-Motion ABI scores are smaller than those for the probe-based ABI scores). If this is the case, then the results of this study could be explained by the Emotion-in-Motion training task producing a greater modification of attentional bias, the Emotion-in-Motion assessment task more sensitively assessing group differences in attentional bias, or both. Future research could circumvent this limitation by employing the same attentional bias assessment approaches for all ABM tasks under evaluation.

A second potential limitation is that this study was carried out on an undergraduate nonclinical participant sample. Although this design allowed us to examine whether the Emotion-in-Motion procedure can induce differential patterns of attentional bias and consequently test the causal impact of these differential patterns of attentional bias on anxiety vulnerability, it does limit conclusion concerning either the acceptability or the efficacy of our novel Emotion-in-Motion ABM approach when used with a clinical sample. Although the complex and dynamic nature of the Emotion-in-Motion task can be expected to enhance face validity of and engagement in the task, future research using clinical cohorts will be necessary to determine whether this novel ABM task is more acceptable to patients than the conventional probe-based training task.

It is also important to consider some potential limitations of using gamification for bias modification. Some of the potential drawbacks are discussed by Boendermaker et al [31]. These authors note that some gamification elements designed to increase motivation (such as visible scores) may be distracting and impair training. Second, implementing intrinsic motivators may be costly and difficult, and the intrinsically motivating value of such elements may vary across individuals. In addition, even if a game is intrinsically motivating, it may need to be combined with a motivation to change in participants before adherence to multi-session training is improved. Most importantly, however, given the strong link between change in attentional bias and change in emotional vulnerability, it is important that in any gamified ABM procedure, the core mechanism underlying ABM (encouraging a change in attentional bias) remains intact [24,59].

### Conclusions

In the meantime, we hope that the Emotion-in-Motion task, which this study has shown to be capable of modifying attentional bias to emotional information and altering anxiety vulnerability as indicated by anxiety reactivity to a stressor, will be of interest and potential value to researchers investigating the potential anxiolytic benefits of directly manipulating maladaptive patterns of attentional bias. To facilitate further research using this task and to encourage independent replication of the findings of this study, we made our Emotion-in-Motion task software freely available [60]. While we look forward to the future evaluation of this novel ABM approach in other cohorts and settings, we also encourage fellow researchers to develop and refine new and innovative ABM paradigms that further enhance our capacity to modify the attentional bias to negative information implicated in anxiety vulnerability and dysfunction. Such continuous improvement in our ABM approaches will optimize the prospect of developing future ABM protocols that prove capable of delivering robust and reliable therapeutic benefits within the clinic.

## Appendix

Multimedia Appendix 1 Further detail on the methods and data handling.

## Abbreviations

ABI: attentional bias index
ABM: attentional bias modification
ANOVA: analysis of variance
