# Supplementary material for: Emotion-in-Motion, a Novel Approach for the Modification of Attentional Bias: An Experimental Proof-of-Concept Study
Source: JMIR Serious Games. 2018 Nov 28;6(4):e10993. doi: 10.2196/10993 (PMC6291684; doi:10.2196/10993)
Supplement: Multimedia Appendix 1 [file games_v6i4e10993_app1.pdf]

Appendix.

Method

Trait anxiety assessment.

The Trait subscale of the State Trait Anxiety Inventory (STAI-T; Spielberger, Gorsuch, Lushene, Vagg, & Jacobs, 1983), was used to assess participants' trait anxiety levels. Total STAI-T scores range from 20-80, with higher scores indicating greater trait anxiety levels. The STAI-T is a commonly used measure of trait anxiety with well-established internal consistency, test-retest reliability and validity across a variety of population groups, including undergraduate students (Barnes, Harp, & Jung, 2002; Spielberger et al., 1983).

Experimental Tasks.

Conventional probe-based attentional bias modification task.

The aim of this task was to induce selective attending to the angry or happy member of face pairs, depending on the assigned training condition. Each trial commenced with the 500ms exposure of a central fixation cross, then two images were presented simultaneously. These two images showed the same person, but in one image displaying a happy emotional expression and in the other image displaying an angry emotional expression. The two images were spaced 13.2cm apart along the horizontal axis, and they were both centrally positioned on the vertical axis. The image pair was presented for 500ms, after which it disappeared and a probe was presented at the former location of one of the images. The probe consisted of two red dots, which were either aligned horizontally (¨) or vertically (:). Participants were required to indicate as quickly as possible whether the dots were aligned horizontally (left mouse click) or vertically (right mouse click). Errors triggered a three second delay before

the next trial started. The probe disappeared when the participant response was made, and the next trial began after a 500ms inter trial interval.

There were 512 trials, with a self-paced pause after every 128 trials. The picture of the angry face was presented equally often on the left and right. In the attend-positive condition the probe was presented in the former location of the image with the happy expression on every trial, and vice versa in the attend-negative condition. Half the trials presented a vertical and half a horizontal probe. The 32 unique training stimulus identities were presented in a random order across the 512 trials, with the constraint that each identity was presented in each of the four combinations of angry picture location (left/right) and probe identity (¨/:) before being presented again.

Conventional probe-based attentional bias assessment task.

The purpose of this task was to assess the impact of the conventional probe-based training task on patterns of attentional bias. To achieve this objective, the training contingency that was present in the training task was removed from this assessment task, which was otherwise identical to the probe-based training task. Specifically, in contrast to the training task, the probe now replaced happy and angry images with equal frequency. The task comprised 128 trials. Each of the 8 unique assessment identities was presented 16 times across these trials, with the constraint that each identity was presented in each of the 8 combinations of angry picture location (left/right), probe location (replace happy/replace angry), and probe identity (¨/:) before being presented again.

To obtain a measure of attentional bias to negative information, an attentional bias index (ABI) was computed by subtracting the reaction time to identify the probe when it was presented in the location of the face displaying a negative emotional expression, from the reaction time to identify the probe when it was presented in the location of the face displaying

a positive emotional expression. A higher positive score on this index reflects greater attention to negative information.

Anxiety reactivity assessment task.

This was the stressor task used in the original ABM study by [1], which has also been used in previous studies evaluating the emotional impact of face-based ABM [2]. The task presented 40 letter strings represented as anagrams to be solved. Twenty of these were extremely difficult but soluble anagrams, in that the letters within each string could be rearranged to spell a legitimate English word. The other 20-letter strings could not be rearranged to form any legitimate English word and so were 'insoluble anagrams'. To increase fear of negative social evaluation, participants were informed that they were to be videotaped while attempting to complete a series of anagrams, and were told that recordings of individuals who did particularly well or particularly poorly would be used as demonstration materials in later laboratory classes. They were advised that they would be shown the anagrams individually on the computer monitor and would have 3 min to complete as many anagrams as possible, by typing in the correct words. Participants were instructed that, after pressing enter to submit their response, they were to press a mouse button to receive the next anagram. If they found an anagram too hard, they were told to leave the response blank and to press a mouse button to receive the next one. The need for both speed and accuracy was emphasized. A video camera then was trained on the participant, and the task commenced. Letter strings from the anagram stimulus set were displayed individually, in a random order, in letters 5 cm high, with a new string appearing each time a mouse button was pressed. Bogus performance feedback was presented on screen throughout, showing that participants' performance was progressively falling further below the "average performance of students who have already completed this task". To measure anxiety reactivity, an analogue mood scale assessing state anxiety was employed before and after the anagram task.

This scale consisted of a 15-cm horizontal line, divided into 60 sections. The anchors of the scale were labelled with the words ‘relaxed’ and ‘anxious’. Participants moved the mouse cursor along the line to a point corresponding to their current level of state anxiety and clicked to register their response. The resulting score was a number between 1 and 60, with higher scores indicating higher levels of state anxiety. The magnitude of increase in state anxiety from before to after the anagram task serves to reveal participants’ anxiety reactivity.

Results

Data handling

In the attentional bias assessment tasks, outlying data points were defined as any reaction time scores (for the conventional dot-probe task), or tracking scores (for the Emotion-in-Motion task), that deviated more than 1.96 SD from the mean scores for each experimental condition. Any such outlying data points were removed prior to computing for each individual the indices of attentional bias to negative information at both the pre-training and post-training time points. Participant outliers were defined as participants who showed an attentional bias index score or state anxiety score at any of the assessment points, or who showed a change in these scores between the respective pre and post assessment points, that deviated more than 1.96 from the mean scores across all participants. Participants identified as outliers according to these criteria were excluded from the statistical analyses.

1. MacLeod, C., et al., *Selective attention and emotional vulnerability: Assessing the causal basis of their association through the experimental manipulation of attentional bias.* Journal of Abnormal Psychology, 2002. **111**(1): p. 107-123.

2. Dandeneau, S.D. and M.W. Baldwin, *The buffering effects of rejection-inhibiting attentional training on social and performance threat among adult students.* Contemporary Educational Psychology, 2009. **34**(1): p. 42-50.
